# Mulibrey nanism and immunological complications: a comprehensive case report and literature review

**DOI:** 10.3389/fimmu.2023.1303251

**Published:** 2023-12-05

**Authors:** Andrea Gazzin, Francesca Pala, Marita Bosticardo, Julie Niemela, Jennifer Stoddard, Eleonora Biasin, Paola Quarello, Diana Carli, Francesca Ferroni, Ottavia M. Delmonte, Davide Montin, Sergio D. Rosenzweig, Francesco Licciardi, Luigi D. Notarangelo

**Affiliations:** ^1^ Laboratory of Clinical Immunology and Microbiology, Immune Deficiency Genetics Section, National Institutes of Health, Bethesda, MD, United States; ^2^ Postgraduate School of Pediatrics, University of Torino, Turin, Italy; ^3^ Department of Laboratory Medicine, National Institutes of Health Clinical Center, Bethesda, MD, United States; ^4^ Pediatric Onco-Hematology, Stem Cell Transplantation and Cellular Therapy Division, Regina Margherita Children’s Hospital, Turin, Italy; ^5^ Immunogenetics and Transplant Biology Unit, Città della Salute e della Scienza University Hospital, Turin, Italy; ^6^ Department of Medical Sciences, University of Turin, Turin, Italy; ^7^ Department of Pediatric Cardiology, City of Health and Science University Hospital, Turin, Italy; ^8^ Department of Public Health and Pediatrics, University of Turin, Pediatria Specialistica U, “Regina Margherita” Children Hospital, Turin, Italy; ^9^ Department of Public Health and Pediatric Sciences, University of Torino, Torino, Italy

**Keywords:** Mulibrey, CD4+ lymphopenia, hypogammaglobulinemia, pericardial constriction, case report

## Abstract

**Introduction:**

Mulibrey nanism (MUL) is a rare disorder caused by *TRIM37* gene variants characterized by growth failure, dysmorphic features, congestive heart failure (CHF), and an increased risk of Wilms’ tumor. Although immune system impairment has been documented in MUL, the underlying mechanisms remain poorly understood.

**Methods:**

We present a case of MUL with progressive lymphopenia and review similar cases from the literature.

**Results:**

Our patient presented with prenatal onset growth restriction, characteristic dysmorphic features, and Wilms’ tumor. She developed progressive lymphopenia starting at 10 years of age, leading to the initiation of intravenous immunoglobulin (IVIG) replacement therapy and infection prophylaxis. Genetic analysis detected a likely pathogenic variant on the maternal allele and copy number loss on the paternal allele in *TRIM37*. Subsequently a cardiac magnetic resonance imaging was conducted revealing signs of pericardial constriction raising concerns for intestinal lymphatic losses. The cessation of IVIG therapy did not coincide with any increase in the rate of infections. The patient exhibited a distinct immunological profile, characterized by hypogammaglobulinemia, impaired antibody responses, and skewed T-cell subsets with an altered CD4+/CD8+ ratio, consistent with previous reports. Normal thymocyte development assessed by artificial thymic organoid platform ruled out an early hematopoietic intrinsic defect of T-cell development.

**Discussion:**

The immunological profile of MUL patients reported so far shares similarities with that described in protein-losing enteropathy secondary to CHF in Fontan circulation and primary intestinal lymphangiectasia. These similarities include hypogammaglobulinemia, significant T-cell deficiency with decreased CD4+ and CD8+ counts, altered CD4+/CD8+ ratios, and significantly modified CD4+ and CD8+ T-cell phenotypes toward effector and terminal differentiated T cells, accompanied by a loss of naïve CD45RA+ T lymphocytes. In MUL, CHF is a cardinal feature, occurring in a significant proportion of patients and influencing prognosis. Signs of CHF or constrictive pericarditis have been evident in the case reported here and in all cases of MUL with documented immune dysfunction reported so far. These observations raise intriguing connections between these conditions. However, further investigation is warranted to in-depth define the immunological defect, providing valuable insights into the pathophysiology and treatment strategies for this condition.

## 1 Introduction

Mulibrey nanism (MUscle-LIver-BRain-EYe nanism, MUL, OMIM #253250) is a rare autosomal recessive disorder caused by biallelic loss of function variants in the *TRIM37* gene (tripartite motif–containing protein 37, OMIM # 605073) (1, 2). The MUL phenotype consists of severe growth failure, dysmorphic facial features, congestive heart failure (CHF) due to constrictive pericarditis and increased risk of Wilms’ tumor (WT) (3, 4). TRIM37 encodes an E3 ubiquitin-protein ligase that belongs to the TRIM-protein superfamily. These proteins play a crucial role in various biological processes such as post-translational modifications, signal transduction, DNA repair, autophagy, and oncogenesis. They have been also implicated in various aspects of immune regulation including antiviral responses, inflammation, and immune cell function (5–8). Few cases of MUL associated to variable degree of cellular and/or humoral immunity impairment have been reported in literature (4, 9–12). Nevertheless, the precise mechanisms by which TRIM37 impacts human immune responses and the underlying pathogenesis of the immunological abnormalities observed in MUL patients remain poorly understood. Although there is acknowledgment of immune system involvement in select MUL cases, a comprehensive understanding of the immunological aspects and their interplay with other clinical features of MUL is still lacking. In this study, we present a case of MUL and provide a thorough examination of its immune dysregulation. Furthermore, we conduct an extensive literature review of documented MUL cases that exhibit immune system involvement. Our primary objective is to contribute to the knowledge concerning the immunological deficits in MUL.

## 2 Case description

### 2.1 Clinical manifestations

**Figure 1:**
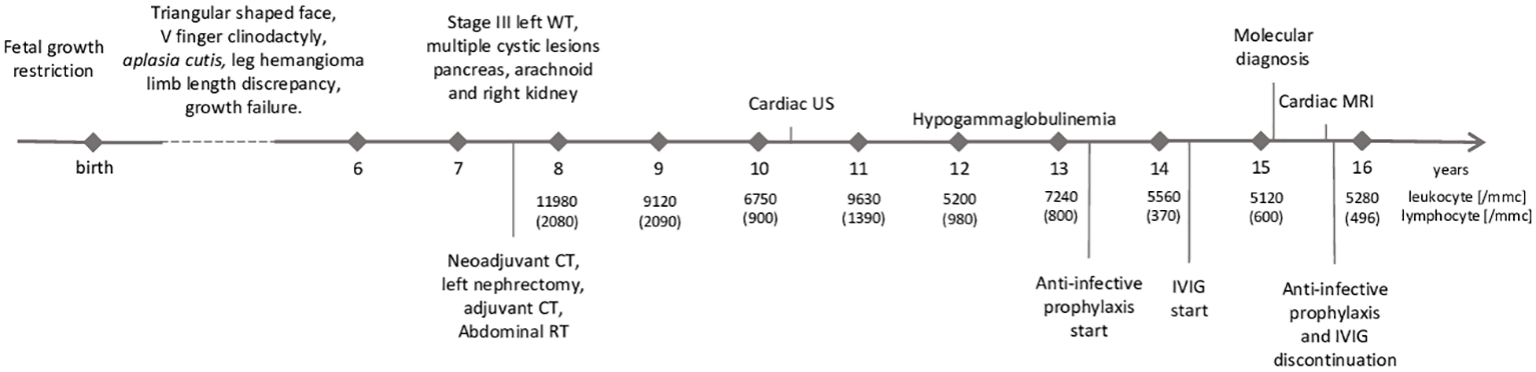
Timeline with relevant data from the patient’s history. WT, Wilms’ tumor; CT, chemotherapy; RT, radiotherapy; US, ultrasound; IVIG,intravenous immunoglobuline; MRI, magnetic resonance imaging.

The proband is a female patient of Italian origin conceived by homologous *in-vitro* fertilization. Upon informed consent, and in accordance to the Helsinki declaration, she was enrolled in protocol 18-I-0128 (NCT03610802 in clinicaltrials.gov) approved by the Institutional Review Board of the National Institutes of Health, Bethesda, MD (USA). Her family history was unremarkable. Pregnancy was uneventful until the seventh month of gestation when signs of fetal growth restriction were detected. The patient was born by caesarean section at 36 weeks’ gestation; birth weight (2200 g, SDs = −0.82), length (45 cm, SDs = −0.66) and cranial circumferences (32.7 cm, SDs = +0.27) were appropriate for gestational age (13). *Aplasia cutis* at vertex and right temporo-parietal scalp areas, linear-shaped achromic patches on right thigh and hemangioma on left and right thigh were evident. The patient also showed mild dysmorphic features, such as triangular-shaped face, mild frontal bossing, slight discrepancy of lower limbs length and circumference. Soon after birth, growth impairment was noted, as height and weight were both markedly below −2 SDs. Growth hormone (GH) replacement therapy was started at the age of 5 years but was soon discontinued due to the diagnosis of WT of the left kidney a few months later. After this diagnosis, the patient started chemotherapy according to the current national protocol. Upon nephrectomy, pathology revealed a stage III WT with no associated nephrogenic rests. Treatment was then completed with adjuvant chemotherapy (including anthracycline at a cumulative dose of 220 mg/m^2^) and abdominal radiotherapy, achieving complete remission. Electrocardiogram (ECG) and cardiac ultrasound (US) performed at follow-up due to anthracycline administration showed mild mitral valve regurgitation with tricuspid regurgitation; left atrium and ventricle sizes were at the upper limit of normal range, and pericardial thickness and morphology appeared regular. Complete blood count performed as part of the oncological follow-up revealed progressive lymphopenia from the age of 10 years (**Figure 1**). Hemoglobin, neutrophil and platelet counts were within normal limits. qPCR for HIV was negative. At the age of 12 years, due to worsening lymphopenia and hypogammaglobulinemia and the absence of anti-Streptococcus pneumoniae and anti-HBs antibodies despite a complete vaccination schedule, intravenous immunoglobulin (IVIG) replacement therapy (0.5g/Kg) and infection prophylaxis with trimethoprim-sulfamethoxazole, azithromycin, and acyclovir were initiated. There have been no major infections in her life. An abdominal magnetic resonance imaging (MRI) scan performed at the age of 13 years showed normal-sized liver and spleen (83 mm). During follow-up, despite regular IVIG infusion every 4 weeks, IgG remained low (360 mg/dL normal value -n.v- 636–1610 mg/dl (14)) with persistently normal IgA and IgM suggesting a chronic IgG loss. This hypothesis was further supported by the progressive decrease of serum albumin levels (3.4 g/dL at 14 years of age and 2.8g/dL 1 year after). Renal function was preserved, and no albuminuria was detected. Liver function tests were remarkable for slight elevation of serum aspartate aminotransferase (49 UI/l) and alanine aminotransferase (38 UI/l), prompting liver elastography that showed increased parenchymal stiffness (15.1 kPa, CAP 207 dB/m). Cardiac MRI was performed and revealed pericardial thickening of 2 mm, paradoxical movement of the intraventricular septum, and enlargement of the inferior vena cava. Atrial and ventricular dimensions and systolic function were preserved. There were no myocardial abnormalities. The patient did not show signs of protein-losing enteropathy (PLE) such as abdominal pain, weight loss, diarrhea, or skin edema. Unfortunately, the patient and her parents refused to undergo a digestive endoscopy.

### 2.2 Genetic findings

**Figure 2:**
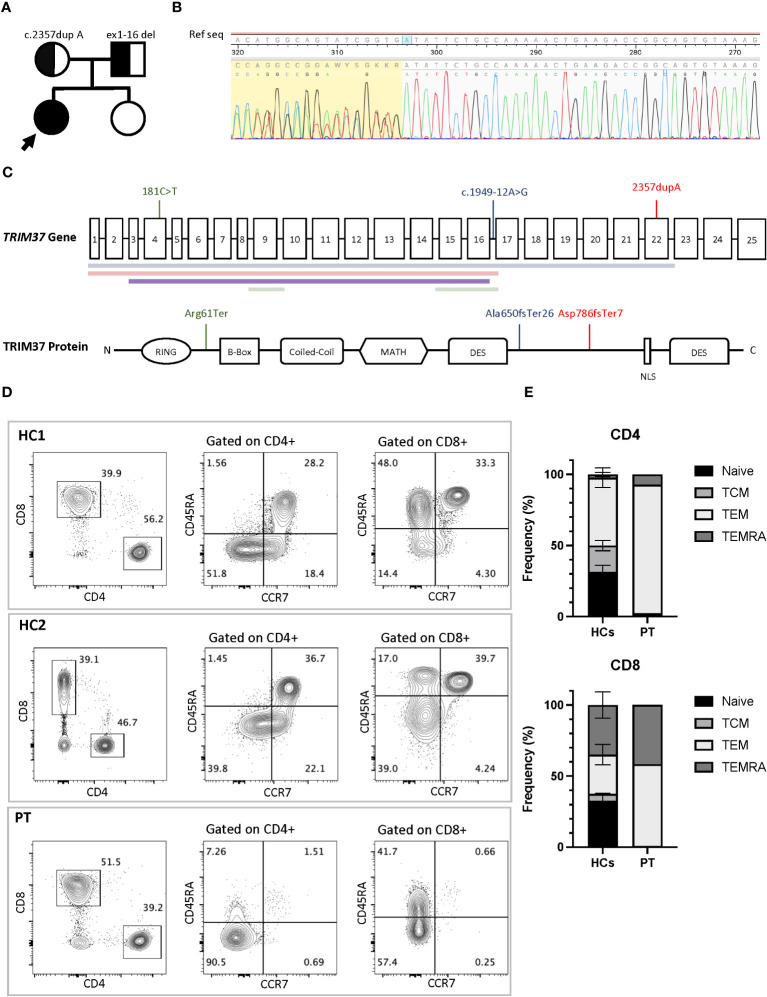
**(A)** Family pedigree with the inheritance pattern. TRIM37 duplication and deletion are showed. **(B)** Sanger sequencing of TRIM37 gene revealed the presence of the monoallelic NM_001320987.3 c.2357dupA (p. Asp786GlufsTer7), a duplication leading to a stop codon seven amino acids downstream. **(C)** Schematic representation of the TRIM37 gene and TRIM37 protein. The positions of mutations and deletions (colored horizontal lines) reported in literature in association with an immunological phenotype are shown in green (Jobic et al) and blue (Bruzzaniti et al) colors. In red is depicted the molecular lesions found in the patient reported in this paper. **(D)** Flow cytometry plots of peripheral blood mononucleate cells from the patient and two healthy controls. CD4+ and CD8+ cells subsets gated on CD3+ cells and T-cell subsets. **(E)** Comparison of T-cell differentiation subsets in patient and controls. HC1, healthy control 1; HCs, healthy control 1 and healthy control 2; PT, Mulibrey patient; TEMRA, terminally differentiated effector memory T cells; TEM, T-effector memory; TCM, T-central memory. Black arrow indicates the proband.

Trio-based Whole Exome Sequencing showed the presence of the monoallelic variant c.2357dupA, p. Asp786GlufsTer7 in the *TRIM37* gene (NM_001320987.3), a duplication resulting in a frameshift and a stop codon 7 amino acids downstream. Sanger sequencing confirmed the presence of the variant in the proband and the mother and the absence in the father (**Figures 2A–C**). A copy number variation on the other allele was suspected since reads mapping showed a nearly half-reduction in reads on exon 13 of *TRIM37* gene in the proband and the father compared to the mother. Chromosomal microarray analysis confirmed the presence copy number loss within chromosome band 17q22 spanning approximately 60 Kb, including exons 1–16 of the TRIM37 gene (min interval 17:57124573–17:57184699; max interval 17:57119399–17:57189128 [GRCh37/hg19]) including *TRIM37* exons 1–16. The deletion was inherited from the father. No significative variants were detected in genes associated with inborn errors of immunity. Chromosomal microarray (CMA) did not identify any copy number changes in this sample that are associated with other known microdeletion or microduplication syndromes. The variant *TRIM37*:c.2357dupA, p.Asp786GlufsTer7 is classified as pathogenic according to the following American College of Medical Genetics and Genomics (ACMG) criteria for single nucleotide variants (15): PVS1 (null variant in a gene where loss of function is a known mechanism of disease), PM2 (extremely low frequency in gnomAD population databases), PP4 (patient’s phenotype or family history is highly specific for the disease), and PM3 (detected in trans with a pathogenic variant). The chr17:57,124,573–57,184,699 [GRCh37/hg19] deletion is classified as likely pathogenic according to the following ACMG criteria for copy number variants (16): 1A (the deleted region contains protein coding or other known functionally important elements), 3A (the deleted region contains 0–24 genes), and 4A (reported proband has a complete deletion of or a LOF variant within gene encompassed by the observed copy number loss and the reported phenotype is highly specific and relatively unique to the gene or genomic region).

### 2.3 Immunological results

**Figure 3:**
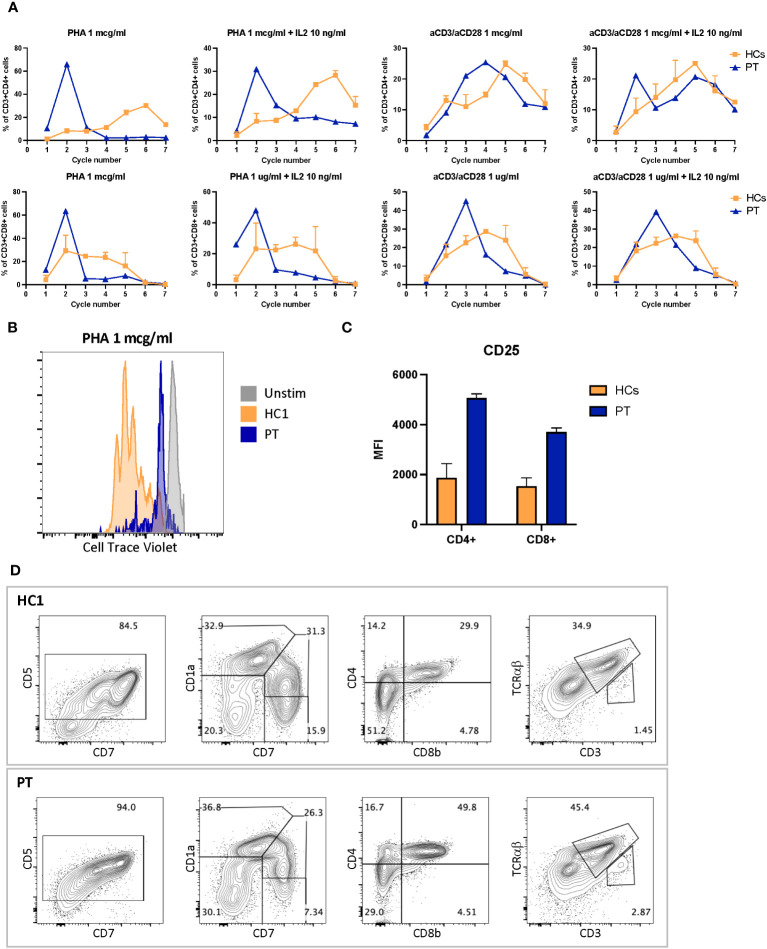
**(A)** CD4+ proliferation assay after 4 days stimulation with phytohemagglutinin 1 mcg/ml or antiCD3 and anti-CD28 1 mcg/ml with or without IL2 10 ng/ml. **(B)** Exemplary cell trace violet dilution after 4 days stimulation with PHA 1 mcg/ml. **(C)** CD25-expression level in CD4+ and CD8+ cells after 4 days stimulation with anti-CD3 1 mcg/ml and anti-CD28 1 mcg/ml. **(D)** Six-week thymocyte development assessed by artificial thymic organoid generated by culturing DLL4-expressing stromal cell line (MS5-hDLL4) with CD34+ cells isolated from peripheral blood cells of the patient compared to healthy control. MFI, mean fluorescence intensity; HC1, healthy control 1; HCs, healthy control 1 and 2; PT, Mulibrey patient; PHA, phytohemagglutinin.

Immunoglobulin quantification performed at 12 years of age showed low IgG level of 443 (n.v 840–1660) mg/dL, with IgG1 256 (n.v. 344–958) mg/dL, IgG2 77 (n.v. 159–406) mg/dL, IgG3 37 (n.v. 35.2–150) mg/dL, and IgG4 34.3 (n.v. 7.86–119) mg/dL. IgA 76 (n.v. 36.4–305) mg/dL and IgM 72 (n.v. 42.4–197) mg/dL were within normal values (14). Anti-*Streptococcus pneumoniae* and anti-HBs antibodies despite a complete vaccination schedule were absent. At 14 years of age peripheral blood lymphocytes were 370/mm^3^. CD3+ lymphocytes were 174/mm^3^ of which CD4 + 73/mm^3^ and CD8 + 88/mm^3^. The immunoglobulin dosage was repeated, confirming low and declining Ig values: IgG levels (398 mg/dL) with IgG1 255 mg/dL, IgG2 57 mg/dL, IgG3 38 mg/dL, IgG4 32 mg/dL, low IgA (76 mg/dL), and IgM (51 mg/dL) levels. Antibody responses against *Streptococcus pneumoniae* and *Clostridium tetan*i were impaired, despite regular vaccination schedule. Flow cytometry analysis of peripheral blood mononucleated cells showed marked reduction of naïve T cells (CD45RA+CCR7+) and increased fraction of effector memory (CD45RA−CCR7−) and terminal effector memory (CD45RA+CCR7−) in both CD3+CD4+ and CD3+CD8+ cells subsets, compared to healthy controls (**Figures 2D, E**). Flow cytometry–based analysis of T-cell receptor (TCR) Vβ repertoires demonstrated a normal distribution among CD4+ Vβ families. However, in the case of CD8+ Vβ families, the distribution exhibited a higher Gini TCR skewing index when compared to controls. The CD4+/CD8+ ratio was 0.8, below the normal range value. B cells count was normal, and switched memory B cells were normally represented. T-cell proliferation ability was assessed upon stimulation with phytohemagglutinin (PHA) and anti-CD3/anti-CD28 antibodies. Proliferation was clearly impaired after PHA and anti-CD3/anti-CD28 stimulation, and addition of interleukin-2 (IL-2) did not rescue proliferation (**Figures 3A, B**). Surface marker analysis upon *in-vitro* activation showed increased expression of the activation marker CD25 when cells were stimulated with anti-CD3/anti-CD28 after 48h, as compared to healthy controls (**Figure 3C**). Nevertheless, the expression of the exhaustion and apoptosis markers (data not shown) did not differ from patient to controls. Since naïve cell subset were reduced, early T-cell development was studied using the artificial thymic organoid (ATO) platform. *In-vitro* T-cell development at 6 weeks of culture appeared normal and comparable to healthy controls (**Figure 3D**), excluding a hematopoietic-intrinsic defect in T-cell development.

A figure or table showcasing a timeline with relevant data from the episode of care

*See* **Figure 1**

## 3 Discussion

In this report, we provide a comprehensive characterization of the immunological phenotype in a Mulibrey patient. Our findings reveal a marked reduction in naïve T cells, an increased proportion of effector memory and terminal effector memory T cells, compromised antibody responses to specific pathogens, a diminished CD4+/CD8+ ratio, and impaired T-cell proliferation and skewed TCR-Vβ repertoire. MUL is a rare growth disorder with prenatal onset caused by biallelic mutations of the *TRIM37* gene on chromosome 17q22 (2). *TRIM37* encodes a 964 amino acids long peroxisomal ubiquitin ligase protein that is ubiquitously expressed. It is part of the RING B-box coiled-coil family of zinc finger proteins and contains the meprin and TRAF homology (MATH) domain. The MATH domain is involved in various processes including protein–protein interaction, signal transduction, protein degradation and, among them, also in the regulation of immune responses (17, 18). TRIM37 participates in antiviral response by restricting the replication of certain viruses, as HIV-1 and influenza A virus, and by promoting the degradation of viral components (6). It has also been linked to inflammation and regulation of inflammatory responses. In tumoral cells and in foam cells, it has been proven that it can modulate the activity of nuclear factor kappa-light-chain-enhancer of activated B cells (NF-κB), a key transcription factor involved in immune and inflammatory pathways, by enhancing its translocation to the nucleus and reducing its ubiquitination (19, 20). Immune system abnormalities in MUL have been reported in literature, but there is significant variability among patients (4, 9–12) (**Table 1**). Frequent respiratory infections were reported in one patient, with benefits after the introduction of IVIG prophylaxis (10), while another patient suffered from pneumonia with sputum culture positive for *Staphylococcus aureus* and *Haemophilus influenzae* (9). These infections were not reported to be lethal or particularly complicated. Humoral immunity dysfunction was reported in all patients, mainly consisting of low IgG (9, 10, 12) with variable increases in IgM (11) or IgA and IgD (10) or IgE (9). Antibody response was impaired in two patients. In particular, in the patient described by Haraldsson et al, antibodies to common respiratory microorganisms and pneumococcal polysaccharides were absent, despite several culture-proven pneumococcal infections; antibodies against diphtheria, tetanus, and poliovirus were absent or transient after normal vaccination and boosters; isohemagglutinin titers and Staphylococcus aureus opsonization were significantly reduced (10). Additionally, Jobic et al. described two families with decreased IgM levels and no vaccine-specific IgG against tetanus despite vaccination (11). On the other hand, the patient reported by Bruzzaniti et al. showed normal IgG titers against rubella and mumps (9). Cellular-immunity involvement was reported in all patients, mostly consisting of T-cell deficiency (9, 10, 12). In-depth T-cell subset analysis was performed in one patient and revealed CD4+ T-cell quantitative and functional defects: both CD4+ and CD8+ T lymphocytes were skewed toward an effector memory phenotype (CD45RA−CCR7−) and double-positive CD4+CD8+ T lymphocytes were increased in frequency. Enhanced expression of T-cell surface activation molecules was demonstrated both in unstimulated and *in-vitro* stimulated conditions. Cell death was not increased, suggesting that the selective reduction of CD4+ lymphocytes was not a result of augmented cell death. CD4+ lymphocyte proliferation was significantly reduced upon TCR stimulation but partially preserved with more physiologic homeostatic stimuli. In addition, *in-vitro* cytokine production by TCR-stimulated T lymphocytes was dysregulated: specifically, CD4+ lymphocytes from the MUL child secreted significantly lower levels of IL-2, IL-4, IL-6, IL-9, IL-13, IL-17A, IL-21, IL-22, interferon (IFN)–γ, tumor necrosis factor (TNF)–α, and TNF-β; on the contrary, they produced higher levels of IL-1, IL-5, IL-8, and IL-31. Moreover, CD8+ lymphocytes produced lower levels of IL-2, IL-8, IL-13, IFN- α, TNF-β, and TNF- γ, while they secreted higher amounts of IL-4, IL-6, IL-9, IL-17A, IL-22, and IL-31, as compared to the healthy controls. Neutrophil, basophil, and monocyte counts were increased, while NK- and B-cell numbers were normal. Thymic MRI was normal (9, 21). Interestingly, in the patient reported by Haraldson et al, T-cell counts were within the normal range, while the B-cell count was low (2% of total peripheral lymphocytes at 2 years of age) (10). Summarizing, hypogammaglobulinemia, with variable impact on different immunoglobulin subclasses, has been consistently documented. However, the involvement of cellular immunity is not consistent. Both B-cell lymphopenia with a normal T-cell count and CD4+ T-cell lymphopenia with a normal B-cell count has been reported. Importantly, for most of the cases reported, detailed studies of the cellular phenotype have not been conducted and, therefore, the dataconcerning cell-mediated immunity from these studies must be interpreted with caution. Consistently, with what reported by Bruzzaniti et al, our patient showed CD4+ T-lymphocyte lymphopenia with altered CD4+/CD8+ ratio and skewed CD4+ and CD8+ T-cell differentiation toward effector and terminal-differentiated T cells together with hypogammaglobulinemia. What is the origin of the immune dysfunction in MUL patients has not been previously demonstrated. Naïve cell reduction may suggest a thymic function impairment or an early hematopoietic intrinsic T-cell development defect. The latter was excluded, as *in-vitro* T-cell differentiation assay in the ATO system was normal. Furthermore a thymic MRI did not show parenchymal structural abnormalities, fibrosis or any other alterations (9). It is noticeable that the immunological findings in MUL patients are similar to the ones showed by children with PLE and Fontan patients with PLE: hypogammaglobulinemia, significant T-cell deficiency with decreased CD4+ and CD8+ count, altered CD4+/CD8+ ratio, and significantly modified CD4+ and CD8+ T-cell phenotype toward effector and terminal differentiated T cells with loss of naïve CD45RA+ lymphocytes (22–24). These quantitative alterations are associated with decreased *in-vitro* lymphocyte proliferative response of T lymphocytes (25). The accumulation of T effector memory lymphocytes and terminally differentiated effector memory T cells is a common feature in patients experiencing recurrent and/or persistent infections, especially of viral origins, such as CMV reactivation (26, 27). However, this is not the case for the patient described, since she did not experience any of those. It is crucial to highlight that that in every case of MUL with immune dysfunction reported in literature, there was evidence of venous congestion in the setting of cardiac dysfunction. Furthermore, in certain cases, a normal immunological finding was previously reported, compatible with a degenerative process (10). In particular, the patient reported by Haraldsson et al. was affected by severely thickened pericardium with markedly impaired diastolic filling of the left ventricle. All patients described by Jobic et al. had hepatomegaly and two had cardiac dysfunction. Bruzzaniti reported a patient diagnosed with constrictive pericarditis with diastolic impairment requiring treatment with furosemide and spironolactone. Finally, the patient presented by Novatcheva et al. was in anasarca status due to constrictive pericarditis. In MUL, heart involvement consists in pericardial thickening without inflammation or effusion and myocardial fibrosis causing diastolic dysfunction and CHF). It occurs in approximately 50% of patients and is the major predictor for prognosis (28, 29). It is worth noting that, in our case and in all the patients with MUL and immune dysfunction reported, there were signs of CHF or constrictive pericarditis. Venous stasis consequent to CHF can lead to leg swelling, jugular venous distension, hepatomegaly and PLE, as showed in patients that underwent Fontan surgery (28, 30). Taken together, these data suggest that the hypercatabolic immunological deficiency in MUL is likely to result from PLE secondary to CHF. However, an additional effect on immune dysfunction in MUL due to TRIM37 depletion in lymphocytes and/or thymic epithelial cells cannot be excluded. What shown in MUL patients may be the result of both increased external losses and inefficient substitution of immune effectors, such as a reduction in thymic output, that is unable to cope with the losses. Furthermore, in spite of the immune deficiency, the patient has displayed no evidence of recurrent or severe infections, demonstrating preserved immune competence. As a result, whether antimicrobial prophylaxis and immunoglobulin replacement therapy are necessary, particularly for patients who do not experience severe or recurrent infections, remains to be demonstrated. Nevertheless, there are limitations to consider in this report. Although the study thoroughly describes the identified immune abnormalities in a MUL case, it does not delve into the specific cellular and molecular pathways affected by TRIM37 mutations. Additionally, although the report suggests constrictive pericarditis as a potential primary cause of immune dysfunction, further research is needed to substantiate this hypothesis. The primary lesson of this report is that the distinctive immunological observations outlined in MUL should prompt consideration of CHF in the presence of constrictive pericarditis. It is acknowledged that diagnosing this condition accurately can be challenging without further investigations such as cardiac MRI or intravenous cardiac catheterization. In fact, our patient was asymptomatic for classic signs of CHF in the first years after the onset of the immune dysfunction, and cardiac US studies performed by trained pediatric cardiologists were negative for signs of venous congestion. Timely identification of constrictive pericarditis and prompt intervention through a total pericardiectomy can halt the advancement of the condition and prevent persistent CHF prior to pericardial adhesion. As observed in similar cases, this approach may also lead to the reversal of PLE and subsequent reconstitution of the immune system, allowing for the discontinuation of IVIG (28, 30). However, it is worth noting that additional, more comprehensive, and focused studies are required to thoroughly characterize the immunological profile of Mulibrey patients.

**Table 1:** Summary of published patients with Mulibrey Syndrome and immune defect.

Authors	*TRIM37* molecular findings (First allele, Second allele)	Infection history	Humoral deficiency	Antibody response	Cellular deficiency	T-cell subsets analysis	T cell stimulation assays	Intravenous Ig replacement therapy	Constrictive pericarditis/diastolic heart failure
Haraldsson et al (10)	N/A	Frequent respiratory tract infections	Low IgG (particularly IgG2 and IgG4) and IgM; High IgA and IgD; Absent IgE	Impaired	B cell lymphopenia	N/A	Normal	+	+
Jobic et al (11)	c.181C>T (*p.Arg61Ter*), Exons 15-16/exon 9 deletion	No	High IgM	Impaired	N/A	N/A	N/A	N/A	+
Bruzzaniti et al (9)	c.1949-12A>G, Exons 1-22 deletion	Pneumonia	Low IgG; Normal IgA and IgM; Slightly increased IgE	Normal IgG titers against rubella and mumps virus	T-cell CD4+ lymphopenia	Increased effector memory (CD45RA-CCR7-) and terminal effector memory (CD45RA+CCR7-) in both CD3+CD4+ and CD3+CD8+ cells	Impaired. Increased cell death upon stimulation	+	+
Novatcheva et al (12)	Homozygous 17q22, Exons 3-16 deletion	N/A	Hypogammaglobulinemia (unspecified)	N/A	T-cell lymphopenia	N/A	N/A	N/A	+
Reported in this article	c.2357dupA (p.Asp786GlufsTer7), Exon 1-16 deletion	No	Low IgG and IgM; Normal IgA	Impaired	T-cell lymphopenia	Increased effector memory (CD45RA-CCR7-) and terminal effector memory (CD45RA+CCR7-) in both CD3+CD4+ and CD3+CD8+ cells subsets	Impaired. Increased CD25 expression upon stimulation.	+	+

N/A, information not available.

“+” means “yes” or “present”.

## 4 Patient’s perspective

From the patient’s perspective, the journey to a MUL diagnosis was an odyssey marked by uncertainty. However, receiving the MUL diagnosis was a turning point. It allowed for the discontinuation of IVIG therapy, sparing the patient from the time-consuming hospital visits for periodic administrations, and enabling the initiation of proper cardiac follow-up. This not only improved the patient’s quality of life but also highlighted the importance of early and accurate diagnosis in reducing the burden of treatment.

## Data availability statement

The original contributions presented in the study are included in the article/supplementary material. Further inquiries can be directed to the corresponding author.

## Ethics statement

The studies involving humans were approved by NIAID IRB (Protocol Number 18-I-0128). The studies were conducted in accordance with the local legislation and institutional requirements. Written informed consent for participation in this study was provided by the participants’ legal guardians/next of kin. Written informed consent was obtained from the individual(s) for the publication of any potentially identifiable images or data included in this article.

## Author contributions

AG: Conceptualization, Investigation, Writing – original draft. FP: Investigation, Writing – review & editing. MB: Investigation, Writing – review & editing. JN: Investigation, Writing – review & editing. JS: Investigation, Writing – review & editing. EB: Writing – review & editing. PQ: Writing – review & editing. DC: Writing – review & editing. FF: Writing – review & editing. OD: Writing – review & editing. DM: Writing – review & editing. SR: Writing – review & editing. FL: Writing – review & editing. LN: Writing – review & editing.
